# Improvement in functional motor scores in patients with non-ambulatory spinal muscle atrophy during Nusinersen treatment in South Korea: a single center study

**DOI:** 10.1186/s12883-024-03725-w

**Published:** 2024-06-20

**Authors:** Jin A. Yoon, Yuju Jeong, Jiae Lee, Dong Jun Lee, Kyung Nam Lee, Yong Beom Shin

**Affiliations:** 1 Department of Rehabilitation Medicine, Biomedical Research Institute, Pusan National University School of Medicine, Pusan National University Hospital, Busan 179 Gudeok-Ro Seo-Gu, Busan, 602-739 Republic of Korea; 2 Department of Rehabilitation Medicine, Pusan National University Hospital, Busan, Republic of Korea

**Keywords:** Spinal muscular atrophy, Nusinersen, Motor skills, Activities of daily living

## Abstract

We analyzed the changes in various motor function scores over a four-year period in patients with non-ambulatory spinal muscular atrophy (SMA) during Nusinersen treatment. Patients underwent Hammersmith Infant Neurological Examination (HINE) or Hammersmith Functional Motor Scale Expanded (HFMSE) before treatment, and approximately every 4 months thereafter. Children’s Hospital of Philadelphia Infant Test of Neuromuscular Disorders (CHOP INTEND) or Children’s Hospital of Philadelphia – Adult Test of Neuromuscular Disorders (CHOP ATEND), Revised Upper Limb Module (RULM), and Motor Function Measure (MFM) were performed based on baseline functional status. Narrative interviews were conducted to explore post-treatment physical improvement regarding activities of daily living (ADLs) and fatigue after ADLs. Based on HFMSE results, 9 patients achieved minimum clinically important differences. Average rates of change (slopes) with corresponding 95% confidence intervals for all assessment tools were in a positive direction. CHOP-INTEND showed the most prominent improvement in children and adolescents followed by HFMSE. Improvements in CHOP-ATEND were most noticeable in adults. Improvements were accompanied by changes in ADLs as observed in the narrative interviews. It is necessary to consider various functional aspects to determine the effectiveness of Nusinersen therapy. The objective assessment of the therapeutic effect of Nusinersen in non-ambulatory SMA requires consideration of functional aspects and the related ADLs.

**Supplementary Information:**

The online version contains supplementary material available at 10.1186/s12883-024-03725-w.

## Introduction

Since the development of disease modifying drugs (DMD) for the treatment of spinal muscular atrophy (SMA) [1, 2], the importance of objective evaluation of symptom improvement to confirm the effectiveness and to guide continued treatment has been emphasized. Nusinersen is an antisense oligonucleotide that modifies SMN2 RNA splicing, thereby increasing protein production [3]. Nusinersen was the first drug to be approved by the United States Food and Drug Administration for the treatment of SMA in pediatric and adult patients [4], and has been administered to eligible patients in South Korea since 2019 based on selective criteria. To maintain the treatment, improvement or maintenance of motor function needs to be demonstrated for two consecutive assessments, compared to the motor function at the time of medication initiation.

According to the recommendations for the diagnosis and management of SMA [5, 6], the Hammersmith Infant Neuromuscular Examination Section 2 (HINE-2) [7] and the Children’s Hospital of Philadelphia Infant Test of Neuromuscular Disorders (CHOP-INTEND) [8] are appropriate for non-sitters, whereas the Hammersmith Functional Motor Scale Expanded (HFMSE) [9], Revised Upper Limb Module (RULM) [10], Motor Function Measure (MFM) [11], and 6 min walk test (6-MWT) [12] are recommended for sitters and above. In addition, the Adult Test of Neuromuscular Disorders (CHOP-ATEND), a modified version of CHOP-INTEND, is a recently developed and recommended tool for chronic adult patients with severe joint contracture and difficulty with wheelchair transfers. In a recent study, this assessment tool yielded the most prominent improvement compared to other measurement tools in chronic non-ambulatory patients [13]. 

During the initial use of Nusinersen, trials only included patients with SMA during infancy [14–16]. However, more recently, the effectiveness of Nusinersen has been demonstrated in patients ranging from infancy to adulthood [13, 17–19]. Chronically affected older patients with SMA vary regarding their functional ability and disease course; in particular, their joint contracture after adolescence requires a different perspective during the functional evaluation compared to the evaluation in younger patients.

As of February 2023, among the 195 individuals who applied for Nusinersen treatment in South Korea, 34 individuals (approximately 17%) discontinued treatment. The cause of medication discontinuation has not been clearly analyzed; however, in addition to expected reasons such as switching to another DMD and drug side effects, it is presumed that the role of insurance coverage related to drug approval in South Korea up to the recent period may have played a role. Recently, CHOP-INTEND and CHOP-ATEND were the only approved motor function assessment tools in South Korea based on reimbursement criteria, in addition to HINE-2 and HFMSE. The appropriate functional assessment tools for SMA patients are recommended based on their SMA type and functionality. In this study, we retrospectively analyzed the changes in various motor function over a four-year period in non-ambulatory SMA during Nusinersen treatment. Based on these results, we aim to discuss areas for improvement and future directions.

## Materials and methods

### Patient selection

This retrospective single-center study included patients with non-ambulatory SMA who underwent motor function evaluation during intrathecal Nusinersen treatment at our medical institution from March 2019 to May 2023. Patients who lacked genetic testing results or received concomitant DMD for SMA other than Nusinersen were excluded from the study.

### Functional motor evaluation

**Fig. 1:**
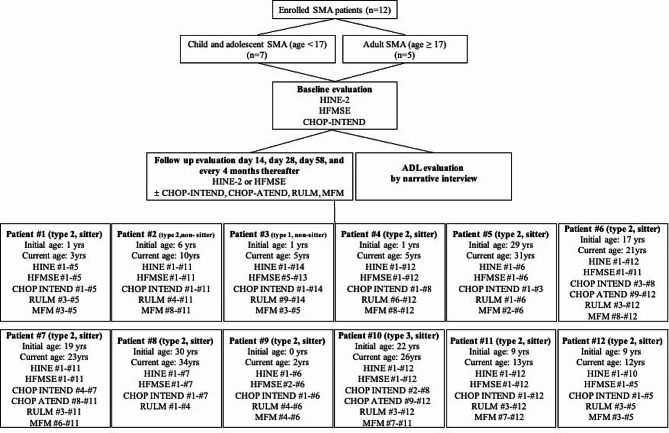
Flow diagram of the study

Patients received an intrathecal loading dose of 12 mg of Nusinersen according to the recommended schedule (baseline, day 14, day 28, day 58, and every 4 months thereafter). The patients underwent HINE or HFMSE aligned with the national reimbursement criteria before treatment and approximately every 4 months after treatment initiation. Additional measurements including CHOP INTEND or CHOP ATEND, RULM, and MFM were performed based on their baseline functional status. (Fig. 1) Additionally, the extent of joint contractures in the cervical and upper/lower joints, which can impact physical function, was assessed.

### Activities of daily living (ADL) evaluation

Additionally, we conducted narrative interviews with patients and caregivers to explore the post-treatment physical improvement focusing on the impact of treatment on ADLs and fatigue after performing ADLs. The interview also assessed the improvement of 10 key ADL domains, including dressing, mobility/transferring, self-care, self-feeding, reaching, picking up and holding objects, physical activity, writing and technology use, social contact/engagement, toileting, and performing work/school activities [20]. To establish a connection between the functional assessment tools and ADL performance, each ADL item was aligned with corresponding items in the functional assessment tool. (Supplementary Table 1)

### Statistical analysis

The model for estimating the overall mean slope included time after Nusinersen initiation with random intercept and slope coefficients. To determine the differences in slopes between the assessment tools, the Wilcoxon signed-rank test was conducted. Additionally, to estimate the fixed effect for the slopes in each tool, a linear mixed model was used. The linear mixed model facilitates the estimation of fixed effects, accounting for repeated measurements within each patient and considering the variation between the different assessment tools. Statistical analysis was performed using the R language version 4.0.2 (R Core Team, 2020, http://cran.r-project.org), and additional packages (stats, lme4, lmerTest, ggplot2). A *p*-value of < 0.05 was considered to be statistically significant. This work was supported by the Department of Biostatistics, Biomedical Research Institute, Pusan National University Hospital.

## Results

A total of 12 patients underwent continuous functional evaluation during Nusinersen treatment. The patients were dived in two groups based on their age at the time of treatment: the children and adolescent (*N* = 7), and adult (*N* = 5) groups. With the exception of one type I and one type III patient, the remaining 10 patients were all type II SMA. Among the 12 patients, 9 (75%) were female. Regarding their functional status, 10 patients (83.3%) were sitters, while 2 patients (16.7%) were non-sitters; in addition, 3 patients (25%) had spinal fusion for scoliosis [21]. The average number of Nusinersen administration was 12 ± 2.93 times followed by functional evaluations. *Three patients were utilizing night-time non-invasive ventilation (NIV) and no additional patients adopted this modality during the course of therapy. During the treatment period, it was not possible for these patients to discontinue the use of ventilators. The mead Force vital capacity (FVC) % pred. at supine position improved from 19.43 to 19.82%, 24.44–30.66% and 21.12–21.50% for patient #5,7,8 respectively.* All patients underwent full oral feeding, and there was no need for nutritional support such as tubal feeding throughout the treatment period. (Table 1) During treatment, there were no observed cases of adverse drug reactions (ADR) or instances of switching to another DMD. In one case (Patient #8), the medication was discontinued after the 7th administration, as there was no observed improvement in serial functional assessment.

**Table 1:** Clinical characteristics of patients

	Total sample (*N* = 12)
Age, mean (min–max), years	15.4 (2–34)
Age at initial treatment, mean (min–max), years	12.0 (0–30)
Sex, n (%)	
Male	3 (25)
Female	9 (75)
SMA type, n (%)	
1	1 (8.3)
2	10 (83.3)
3	1 (8.3)
SMN2 copy number	
2	0
3	12 (100%)
4	0
Functional status, n (%)	
Non–sitter	2 (16.7)
Sitter	10 (83.3)
Spinal fusion, n (%)	
Yes	3 (25)
No	9 (75)
Ventilator apply, n (%)	
Yes	3 (25)
No	9 (75)

Spinal muscular atrophy, SMA; Survival of motor neuron 2, SMN2

The frequency and timing of functional assessments conducted during each patient’s course of treatment are summarized in Fig. 1. Baseline scores and the number of initial evaluations for the various functional assessment tools are presented in Table 2. Based on the improvement of HFMSE, 9 patients achieved minimal clinically important difference (MCID; HFMSE score change ≥ 3 compared to the baseline value) [9, 22], while the 3 remaining patients, all adults, did not achieve MCID (Patients #5, 7, and 8). Patient #8 did not show improvement in any of the assessment tools after Nusinersen treatment, and therefore they were excluded from the selection criteria after 10 sessions. On the other hand, patient #7, who did not achieve an MCID based on HFMSE, showed score improvement in other assessment tools including 7 points for CHOP-INTEND, 5 points for CHOP ATEND, and 3 points for RULM.

**Table 2:** Baseline scores of the functional assessment tools

Patient	Sex, age (years)	type	HINE	HFMSE	CHOP–INTEND	RULM	MFM	CHOP–ATEND
1	F,3	2	9 (#1)	8 (#1)	36 (#1)	13 (#3)	33 (#4)	NT
2	F,10	2	5 (#1)	0 (#1)	23 (#1)	11 (#4)	24 (#8)	NT
3	F,5	1	5 (#1)	7 (#5)	33 (#1)	18 (#9)	NT	NT
4	F,5	2	18 (#1)	28 (#1)	56 (#1)	25 (#6)	56 (#8)	NT
5	F,31	2	12 (#1)	7 (#1)	37 (#1)	17 (#1)	40 (#2)	NT
6	M,21	2	11 (#1)	7 (#1)	39 (#1)	17 (#3)	49 (#8)	36 (#9)
7	F,23	2	10 (#1)	6 (#1)	38 (#1)	15 (#3)	36 (#6)	28 (#8)
8	M,34	2	3 (#1)	0 (#1)	6 (#1)	0 (#1)	NT	NT
9	M,2	2	11 (#1)	5 (#1)	38 (#1)	14 (#4)	31 (#4)	NT
10	F,26	3	6 (#1)	3 (#1)	34 (#1)	16 (#3)	36 (#1)	33 (#9)
11	F,13	2	12 (#1)	10 (#1)	37 (#1)	18 (#3)	42 (#7)	NT
12	F,12	2	9 (#1)	10 (#1)	33 (#1)	17 (#1)	44 (#5)	NT

Hammersmith Infant Neuromuscular Examination Section, HINE; Hammersmith Functional Motor Scale Expanded, HFMSE; Children’s Hospital of Philadelphia Infant Test of Neuromuscular Disorders, CHOP-INTEND; Revised Upper Limb Module, RULM; Motor Function Measure, MFM; Children’s Hospital of Philadelphia – Adult Test of Neuromuscular Disorders, CHOP-ATEND

#, number of initial evaluations after initial Nusinersen treatment

**Fig. 2:**
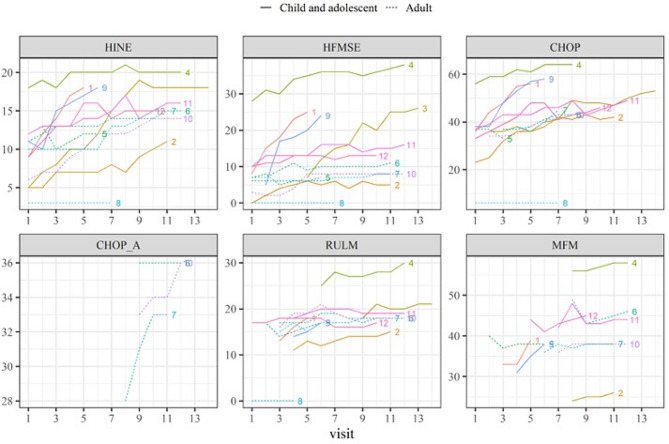
Scatterplot showing the trajectory of each patient based on various assessment tools

**Fig. 3:**
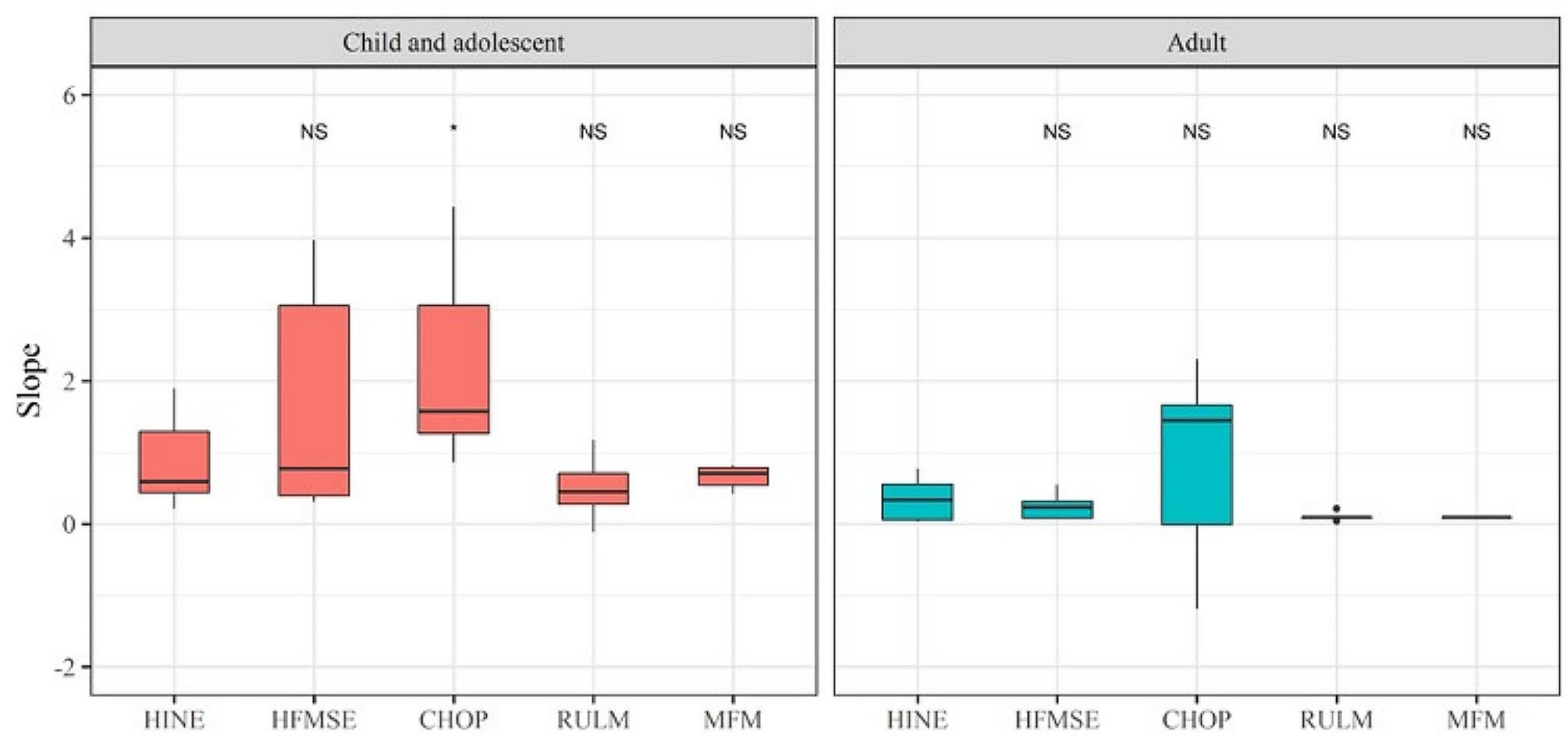
Box plot of slopes showing average annual rate of change using linear mixed model

Average rates of change (slopes) with corresponding 95% confidence intervals for all assessment tools were in a positive direction, although some were accompanied by wide CIs. In the children and adolescent group, the most prominent improvement was observed with the CHOP-INTEND (mean slope = 2.215 points/year, 95% CI 0.977–3.527, *p* = 0.014), followed by HFMSE (mean slope = 1.706 points/year 95% CI 0.376–3.066, *p* = 0.040). (Fig. 2) Improvement of CHOP-ATEND scores were most noticeable for adult patients and exhibited statistical significance (mean slope = 0.912 points/year, 95% CI 0.345–1.583, *p* = 0.013). (Table 3) A comparison of the differences in slopes between the assessment tools revealed that CHOP-INTEND exhibited significantly higher slopes compared to HINE, RULM, and MFM (*p* < 0.05) in the children and adolescent group. (Fig. 3; Supplementary Fig. 1) Improvements in scores were accompanied by changes in ADLs, as observed in the narrative interviews associated with ADLs; their relation to the functional assessment tools are summarized in Table 4.

**Table 3:** Linear mixed analysis to estimate the mean change of each outcome

Group	Outcome	Coefficient	SE	df	*P*–value	95% CI
Child & adolescent	HINE	0.880	0.259	4.858	0.020	(0.353–1.445)
	HFMSE	1.706	0.642	5.684	0.040	(0.376–3.066)
	CHOP–I	2.215	0.607	5.088	0.014	(0.977–3.527)
	RULM	0.493	0.205	2.199	0.127	(0.095–0.994)
	MFM	0.659	0.321	2.835	0.138	(0.025–1.560)
Adult	HINE	0.348	0.159	3.845	0.096	(–0.003–0.681)
	HFMSE	0.248	0.122	3.471	0.123	(–0.039–0.493)
	CHOP–I	0.845	0.698	3.180	0.308	(–0.696–2.317)
	CHOP–A	0.912	0.290	8.224	0.013	(0.345–1.583)
	RULM	0.104	0.088	2.967	0.324	(–0.096–0.287)
	MFM	0.091	0.182	18.668	0.621	(–0.261–0.469)

Hammersmith Infant Neuromuscular Examination Section, HINE; Hammersmith Functional Motor Scale Expanded, HFMSE; Children’s Hospital of Philadelphia Infant Test of Neuromuscular Disorders, CHOP-INTEND; Revised Upper Limb Module, RULM; Motor Function Measure, MFM; Children’s Hospital of Philadelphia – Adult Test of Neuromuscular Disorders, CHOP-ATEND

**Table 4:** Cases of narrative interview associated with ADLs and the relation to function assessment tools

	Patient #1 (age: 3yrs)	Patient #4 (age: 5yrs)	Patient #7 (age: 23yrs)	Patient #12 (age 12yrs)
Related improved items	HFMSE #3,4 CHOP–INTEND #2,3,8,9 MFM #17 RULM D, G,J, K	HFMSE #2,6,9,10,21,22,23 CHOP–INTEND #3,10,11 MFM #17,20 RULM H, O,R	HFMSE #3 CHOP–INTEND #1,9,10,11,13 MFM #17 RULM G, H,I	HFMSE #3 CHOP–INTEND #2,9,11,15 MFM #19, RULM H
Improved ADLs				
Self–care	Squeeze toothpaste and brush teeth Rub hands with soap Comb bangs	Operate a faucet Squeeze toothpaste and brush teeth Mouthwash cup Comb back of hair Dry body using a towel	Squeeze toothpaste Brush teeth using electric toothbrush	Toothbrushing Wash own face Comb hair Doing make–up
Self–feeding	Open and close lids Use a spoon Transfer water into cup	Open and close lids Use a spoon Transfer water into cup	Drink from a cup Use a spoon	Preparing meal Use a spoon and folk
Toileting	Sit on the toilet Tear toilet pater Flush the toilet	Lift the toilet cover Sit on the toilet Tear, fold and discard toilet paper,	Dependent to caregiver	Lift the toilet cover Sit on the toilet, Tear, fold and discard toilet paper
Dressing	Take off socks and shoes	Put on and take off tops and bottoms Wear and remove shoes Operate zippers	Dependent to caregiver	Take out an unfold clothes Put on and take off bottoms with support
Mobility	Dependent to caregiver	Dependent to caregiver	Dependent to caregiver	Electric wheelchair use

Hammersmith Infant Neuromuscular Examination Section, HINE; Hammersmith Functional Motor Scale Expanded, HFMSE; Children’s Hospital of Philadelphia Infant Test of Neuromuscular Disorders, CHOP-INTEND; Revised Upper Limb Module, RULM; Motor Function Measure, MFM; Children’s Hospital of Philadelphia – Adult Test of Neuromuscular Disorders, CHOP-ATEND

## Discussion

In this study, we performed long-term evaluation using functional assessment tools and assessment of ADLs in patients of different age groups during Nusinersen treatment. This study examined the extent of improvement among different assessment tools and the presence of concomitant improvement of ADLs related to the improved items of the assessment tools. Nusinersen is effective in improving motor function in patients with SMA [4, 23, 24]. The appropriate functional assessment tools for SMA patients are recommended based on their SMA type and functionality. However, this study stands out as the first to present results from serial evaluations using various assessment tools on the same patients, particularly those with non-ambulatory SMA. Additionally, the narrative interview results regarding improvement in ADLs following Nusinersen treatment, as indicated in this study, suggest the importance of selecting objective assessment tools for SMA patients. Moreover, they highlight the potential and necessity of assessing improvement in ADLs, which may be challenging to quantify numerically, as an additional indicator for evaluating the effectiveness of therapy.

Improvement in motor function after treatment should be assessed using appropriate functional assessment tools. While HFMSE is commonly used, CHOP-INTEND is additionally recommended for detecting improvement in younger children and in patients with advanced weakness. In addition, CHOP-ATEND or assessments like RULM [25] and MFM [26] can be performed to compensate for low HFMSE scores. Specifically in older patients, a standardized protocol for evaluating neuromotor function cannot be universally applied to the entire patient spectrum [27, 28]. In a recent study based on Real-World Data, Nusinersen was found to be effective for SMA type II patients, as demonstrated by the HFMSE results. This trend aligns with the findings of our study. However, as highlighted in this study [29], the assessment of improvement in SMA type III or adult patients may vary depending on the choice of evaluation tools. In patients who may show floor effect of other assessment tools, CHOP-ATEND can be additionally applied to assess the broader range of motor improvement [13]. According to the results of our study, improvement of CHOP-ATEND scores were most noticeable for adult patients and exhibited statistical significance.

None of the scales currently in use for patients with SMA are designed to assess everyday living functions, gross motor functions, and small muscle skills together, and none can be applied to all individuals with SMA [6]. Based on the improvement trends observed in the evaluation tools presented in this study, non-sitter patients were unable to attempt any of the items presented in HINE and HFMSE, such as crawling, standing, and walking. HFMSE exhibited improvement trends during the early stage of treatment, but the non-ambulatory patients could not perform most items above item #18 (performed in standing position), which could lead to floor effect after a certain number of Nusinersen sessions. Additionally, as mentioned above, there are limitations in using HINE or HFMSE for evaluating upper extremity improvement in patients with non-ambulatory SMA, particularly those in the adolescent and adult age groups who perform ADLs in a wheelchair-bound situation. Therefore, various functional measures should be evaluated together to confirm functional improvement and overcome the limitations of HINE and HFMSE.

As demonstrated in this study, the slope of improvement over four years was most prominent in CHOP-INTEND for younger patients and in CHOP-ATEND for adult patients, capturing a broader range of motor abilities. CHOP-ATEND was especially beneficial for adult patients with severe contractures who cannot lie prone or are difficult to evaluate outside of a wheelchair. In cases where there are limitations in evaluating specific items with CHOP-INTEND due to joint contracture in adolescents and adults, CHOP-ATEND can be used as an alternative evaluation tool. As an example, a patient in our study (Patient #7) who did not achieve MCID for HFMSE showed score improvement with other assessment tools. Notably, the items that showed improvement for this patient in CHOP-INTEND and ATEND were shoulder flexion, elbow flexion, knee extension, and head control with wheelchair sitting. However, these improvements were difficult to detect using HFMSE. In addition, the results of upper extremity function assessment tools presented in the study revealed that 7 out of the 12 patients showed continuous score improvements in RULM or MFM. Although the MCIDs for these assessment tools are not specified, the items showing improvement with these tools were not detected in other assessments. These improvements were often related to actions directly associated with ADLs that patients perform. Moreover, given that previous reports have correlated items of MFM with related ADLs [11, 20] for patients above the adolescent age with multiple contractures, the focus after Nusinersen treatment should be on the improvement of fine motor function, which contributes to improvements in related ADLs. These aspects require proper evaluation using upper extremity assessment tools other than HINE-2 or HFMSE.

Indeed, the ultimate goal of Nusinersen therapy is not only to improve scores on simple motor function assessment tools but also to enable patients to perform actions they were previously unable to do, and to sustain activities they could previously perform without undue fatigue. *Current drug approval criteria mostly consider only motor function improvement of SMA patients. To determine the long-term effectiveness of the medication, it is important to consider that these motor function scales may exhibit a ceiling effect at some point. Therefore, in addition to assessing motor function, tools for evaluating respiratory function, fatigue and ADLs based on SMA type and age should be considered as criteria for determining the continuation of the treatment.* Moreover, commonly used motor function assessment tools have limitations in capturing detailed improvement in ADLs, as described in cases of our narrative interviews. Therefore, during evaluating improvement in motor functions, it is essential to consider improvements in fatigue as another evaluation tool [30]. *In addition, although we did not focus on bulbar function including oral communication or swallowing dysfunction in this study, bulbar function should be monitored as a crucial aspect especially in type I and II patients. The currently used functional scales do not include this domain. Therefore, quantitative functional assessment related to communication and eating, along with qualitative interview about related ADL improvement should be considered as complementary measurement.* Evaluating individual improvements in ADLs is crucial, as well as providing opportunities for patients to attempt and perform activities they had not tried before. Based on the association between the items of motor assessment tools and ADLs outlined in this study, it is meaningful to assess the improvement in ADL performance related to each patient’s score enhancement. Additionally, providing patients with the opportunity to practice and attempt actions that are deemed feasible in their environment can be highly beneficial. For instance, in the case of patient #4 in this study, during the course of treatment the RULM assessment showed that the patient achieved full scores for the items for shoulder flexion, abduction, tracing path, and tearing paper (Items C, H,O, R). Furthermore, through patient interviews and by referring to Supplementary Table 1, improvements in assessment-based related ADLs were noted: the patient reported being able to independently operate a faucet, open and squeeze toothpaste, comb hair, open food container lids, and put on upper garments. Additionally, the patient mentioned practicing to perform additional ADL tasks that they had not previously attempted, and these tasks would be continuously monitored. As such, patients can experience meaningful improvement in their overall functional abilities, and can achieve a better quality of life.

The limitations of this study include its small sample size, which is attributed to the nature of this rare disease, and the analysis of long-term data obtained from a single medical institution. This study focused on tracking and observing the functions of 12 non-ambulatory SMA patients over an extended period using various functional assessment tools at a single tertiary hospital. Although several functional assessment tools tailored to SMA patients have been proposed to date, no studies have simultaneously examined the trend of changes in various assessment tools in individual patients. The results presented in this study, illustrating the long-term changes in motor function during Nusinersen treatment of non-ambulatory SMA patients, emphasize the need for tailored evaluation based on function. The findings also highlight the importance of objectively assessing the continuity of treatment, and suggest that the evaluation should not be limited to assessment tools alone but should also encompass changes in ADLs.

Additionally, in this study, the appropriateness of the commonly used exercise evaluation tools was assessed focusing on non-ambulatory SMA patients. Unfortunately, we were unable to conduct an analysis on a diverse range of patients with various SMA types and functions. However, the study consisted mostly of SMA type II sitters. In addition, among the two non-sitter patients, one demonstrated functional improvement to partial sitting during the treatment process.

Lastly, in this study, some evaluation tools were not performed in all visits. Specifically, for CHOP-A, RULM, and MFM, the initial assessment time was determined as the point at which the evaluation was deemed feasible, and follow-ups were conducted accordingly. Consequently, there were fewer initial assessment points and repetitions for these assessment tools compared to HINE, HFMSE, and CHOP INTEND. To address these considerations, we conducted analyses using linear mixed models to examine the differences among the tools and to assess the slopes resulting from repeated measurements.

## Conclusion

This study provided insights into the improvement and patterns of change of various functional assessment tools in non-ambulatory SMA after Nusinersen treatment. As demonstrated by our findings, it is necessary to consider various functional aspects to determine the effectiveness of Nusinersen therapy. Additionally, focusing on the improvement of related ADLs is necessary to objectively assess the therapeutic effect and ensure meaningful improvement in the patients’ daily lives.

### Electronic supplementary material

Below is the link to the electronic supplementary material.

Supplementary Material 1

Supplementary Material 2

## Data Availability

No datasets were generated or analysed during the current study.
