# Supplementary material for: Improvement in functional motor scores in patients with non-ambulatory spinal muscle atrophy during Nusinersen treatment in South Korea: a single center study
Source: BMC Neurol. 2024 Jun 20;24:210. doi: 10.1186/s12883-024-03725-w (PMC11188501; doi:10.1186/s12883-024-03725-w)
Supplement: Supplementary file 1 — Supplementary Material 1 [file 12883_2024_3725_MOESM1_ESM.docx]

Supplementary Table 1. Activities of daily living (ADL) sheet in patients with spinal muscular atrophy

Date: ______________

Name: ______________

Sex/age: ______________

| **Dressing** | | **MFM-32** | **HFMSE** | **RULM** | **CHOP INTEND** |
| --- | --- | --- | --- | --- | --- |
|  | Put on/take off the bag | 15 |  | A, F, O, P, Q, R, S, T | 1, 8, 9 |
|  | Deal with zippers, buttons, and ties | 15, 17, 20 |  | A, D, E, F, H | 1, 8, 9 |
|  | Put on/take off T-shirts | 5, 15 |  | A, F, O | 1, 8, 9, 13 |
|  | Put on/take off pants | 3, 6, 26 | 21, 22, 25, 26, 27, 28 |  | 1, 2, 5, 10, 11 |
|  | Put on/take off socks and shoes | 3, 4 | 21, 22, 23, 24, 25, 26, 27, 28 |  | 1, 2, 11 |
|  | Tie/untie shoelaces | 3, 4 | 21, 22, 23, 24, 25, 26, 27, 28 |  | 1, 2, 11 |
|  | Take clothes out of/ put clothes in a wardrobe/drawer | 10, 16 | 3, 4 | A, F, N, O, P, Q, R, S, T | 1, 8, 9 |

| **Mobility/transfer** | | **MFM-32** | **HFMSE** | **RULM** | **CHOP INTEND** |
| --- | --- | --- | --- | --- | --- |
|  | Lift your head off the bed/pillow | 1, 2 | 17 |  | 4, 12, 14 |
|  | Change position by turning and moving | 7 | 5, 6, 7, 8, 9, 21, 22 |  | 2, 6, 7, 11 |
|  | Prevent asphyxiation in a prone position (by supporting with arms) | 7 | 6, 7, 11, 12, 13, 15 |  | 1, 6, 7, 15 |
|  | Get up from and lie on the bed/floor | 8, 9, 10, 13 | 1, 2, 4, 5, 6, 7, 8, 9, 10, 14 |  | 1, 2 |
|  | Sit with both legs stretched out in front | 10, 13, 23 | 2, 5, 6, 7, 8, 9 |  | 2, 10 |
|  | Sit on a flat bench mat/stool | 9, 10, 13, 23 | 1, 2, 5, 6, 7, 8, 9 |  | 2 |
|  | Move on the floor | 10 | 5, 6, 7, 8, 9, 15, 16 |  | 1, 2, 6, 7, 11 |
|  | Crawl on all fours | 7 | 5, 6, 7, 8, 9, 11, 15, 16 |  | 1, 2, 12, 15 |
|  | Stand up when falling/from sitting on the floor | 11 | 25, 26 |  | 1, 2, 10 |
|  | Keep standing | 25 | 18, 19 |  | 2, 10 |
|  | Sit on a chair if tired or unstable from standing | 12 | 27, 28 |  | 2, 10 |
|  | Stand up from sitting on a chair | 24 | 28 |  | 2, 10 |
|  | Adjust foot position in a wheelchair |  | 21, 22 |  | 2, 5, 10, 11 |
|  | Assist when moving from wheelchair to bed/bathroom | 10, 13, 24 | 21, 22, 23, 24, 25, 26, 27 |  | 1, 2, 8, 9, 11 |
|  | Go down from the sofa/wheelchair to the floor | 10, 13, 24 | 5, 6, 7, 8, 9, 10, 15, 23, 24, 27 |  | 1, 2, 8, 9, 11 |
|  | Go up to the sofa/wheelchair from the floor | 10, 11, 13, 24 | 14, 15, 23, 24, 25, 26 |  | 1, 2, 8, 9, 11 |
|  | Operate a joystick on an electric wheelchair | 17, 18, 19, 22, 23 |  | C, K, L, M | 1, 3 |

| **Personal hygiene** | | **MFM-32** | **HFMSE** | **RULM** | **CHOP INTEND** |
| --- | --- | --- | --- | --- | --- |
|  | Comb hair | 15 | 3, 4 | A, B, D, E, O | 1, 3, 8, 9 |
|  | Wear a mask/glasses/earrings/hairband | 15, 17 | 3, 4 | A, B, D, E, F, O | 1, 3, 8, 9 |
|  | Blow nose and wipe | 15, 17 | 3, 4 | A, B, D, E, F, O | 1, 3, 8, 9 |
|  | Do makeup | 15, 17 | 3, 4 | A, B, D, E, F, O | 1, 3, 8, 9 |
|  | Use a full-length mirror | 25 | 18, 19, 20 |  | 2 |
|  | Raise/lower the faucet in the sink and turn the faucet | 16, 17, 20, 21, 23 |  | A, B, E, G | 1, 3, 9 |
|  | Squeeze toothpaste | 15, 16, 17, 20 |  | A, B, D, G | 1, 3, 8, 9 |
|  | Brush teeth | 15, 17 | 3 | A, B, D, E, G, J | 1, 3, 8, 9 |
|  | Rinse the mouth with a mouthwash cup |  | 3 | A, B, J | 1, 3, 8, 9 |
|  | Lather hands with soap, wash hands, and dry hands with a towel | 15, 16, 17 |  | A, E, D | 1, 3, 8, 9 |
|  | Lather and rinse face and dry face with a towel | 15, 17 | 3, 4 | A, E, F, O | 1, 3, 8, 9 |
|  | Move into the bathtub | 13, 26 | 23, 24, 25, 26, 27 |  | 2, 10, 11 |
|  | Sit in the bathtub | 10, 13, 23 | 1 |  | 2, 10, 11 |
|  | Take out bath products | 16, 17 |  | A, B, D, E, F, N, O, P, Q, R, S, T | 1, 3, 8, 9 |
|  | Pump bath liquids | 18, 22, 23 |  | B, D, E, F, G | 1, 8, 9 |
|  | Wash hair, take the shampoo in hand, and bring it to head | 15 | 3, 4 | A, O | 1, 8, 9 |
|  | Lather arms/ body | 5, 15 | 3 | A, E, F, G, O | 1, 3, 8, 9 |
|  | Lather legs/feet | 3, 27 | 21, 22, 23, 24, 25, 26, 27, 28 |  | 1, 2, 3, 11 |
|  | Take a bath yourself | 15, 27 | 18, 19, 20, 25, 26, 27, 28 | A, O | 1, 2, 3, 8, 9, 10, 11 |

| **Eating** | | **MFM-32** | **HFMSE** | **RULM** | **CHOP INTEND** |
| --- | --- | --- | --- | --- | --- |
|  | Drink water with a straw while lying down | 1, 2 | 17 |  | 4, 12, 14 |
|  | Drink a glass of water while holding a cup in hand |  | 3, 4 | A, J | 1, 3, 8, 9, 15 |
|  | Put hands on the table | 15, 16, 23 | 3, 4 | A, B, F | 1, 8, 9 |
|  | Bring/push aside side dishes on the table | 16, 17, 23 | 3, 4 | A, B, D, E, K, L, M | 1, 3, 8, 9 |
|  | Scoop up food | 5, 15, 16, 23 | 3, 4 | A, B, E, F, J, K, L, M | 1, 3, 8, 9 |
|  | Use a spoon and chopsticks/fork | 16, 17, 21, 23 | 3, 4 | A, B, D, J, K, L, M | 1, 3, 8, 9 |
|  | Bring food to the mouth | 15, 16, 17, 23 | 3, 4 | A, B, D, F, J, K, L, M | 1, 8, 9 |
|  | Open the lid of the side dish container | 20, 23 |  | B, C, H, I | 1, 3 |
|  | Season with salt/pepper, etc. | 15, 16, 17, 21, 23 | 3, 4 | A, B, D, F, K, L, M | 1, 3, 8, 9 |
|  | Put and mix rice/soup | 16, 23 | 3, 4 | B, K, L, M | 1, 3, 8, 9 |
|  | Pour water into a cup | 15 | 3, 4 | A, J, K, L, M | 1, 3, 8, 9 |
|  | Take food out of the refrigerator/cupboard | 10, 15, 16 |  | A, F, N, O, P, Q, R, S, T | 1, 3, 8, 9 |
|  | Use a gas stove/microwave | 10, 15, 16, 17, 20 |  | A, F, N, O, P, Q, R, S, T | 1, 3, 8, 9 |
|  | Eat in a sitting position | 13, 14, 16, 17, 23 | 1, 2, 3, 4 | B | 12, 15 |
|  | Stand up from a chair after eating | 24 | 25, 26 |  | 2, 10 |

| **Reaching for and picking up things** | | **MFM-32** | **HFMSE** | **RULM** | **CHOP INTEND** |
| --- | --- | --- | --- | --- | --- |
|  | Pick up small things such as coins | 17 |  | D | 1, 3 |
|  | Count banknotes/handouts | 17, 20 |  | D, H, I | 1, 3 |
|  | Organize things on the desk | 16, 17, 23 | 3, 4 | B, D, E, K, L, M | 1, 3, 8, 9 |
|  | Take out/organize things on a shelf/bookshelf | 10, 16, 23 | 3, 4 | E, F, N, O, P, Q, R, S, T | 1, 3, 8, 9 |
|  | Pick up things on the floor | 27, 32 | 23, 24, 25, 26, 27, 28 |  | 1, 2, 3, 8, 9 |

| **Writing and using the device** | | **MFM-32** | **HFMSE** | **RULM** | **CHOP INTEND** |
| --- | --- | --- | --- | --- | --- |
|  | Paint and write | 16, 17, 18, 19, 22, 23 |  | B, C | 1, 3 |
|  | Use a touchscreen such as a cellphone/tablet PC | 16, 18, 19, 22 |  | C, F, G | 1 |
|  | Manipulate the keyboard/mouse | 16, 17, 18, 19, 22, 23 |  | C, F, G | 1 |
|  | Put the backpack and tablet PC on the desk | 23 |  | B, N | 1, 3, 8, 9 |
|  | Turn on and off the desk lamp on the desk | 10, 16, 18, 19, 22, 23 |  | B, F, G | 1, 8, 9 |
|  | Press the call bell in case of emergency | 10, 16, 18, 19, 22, 23 |  | B, F, G | 1, 8, 9 |

| **Physical activity** | | **MFM-32** | **HFMSE** | **RULM** | **CHOP INTEND** |
| --- | --- | --- | --- | --- | --- |
|  | Walk | 26, 28, 29, 30 | 20 |  | 2 |
|  | Run | 26, 30 |  |  | 2 |
|  | Hop and jump over obstacles | 26, 31 | 29, 30, 31, 32, 33 |  | 2 |
|  | Go up and down stairs | 26, 32 | 30, 31, 32, 33 |  | 2 |
|  | Resistance strength training on the lower extremity | 26, 27, 28, 31, 32 | 28, 29, 30, 31, 32, 33 |  | 2 |
|  | Dance | 26, 27, 28, 29, 30, 31, 32 | 28, 29, 30, 31, 32, 33 |  | 2 |

| **Social participation** | | **MFM-32** | **HFMSE** | **RULM** | **CHOP INTEND** |
| --- | --- | --- | --- | --- | --- |
|  | Participate in conversation | 14 |  |  | 12 |
|  | Look around | 1, 2 |  |  | 12, 14, 15 |
|  | Embrace | 5 |  |  | 1, 8, 9 |

| **Toileting** | | **MFM-32** | **HFMSE** | **RULM** | **CHOP INTEND** |
| --- | --- | --- | --- | --- | --- |
|  | Lift the buttocks when changing a diaper | 6 | 21, 22 |  | 2, 11 |
|  | Keep a sitting position on the toilet | 9, 13, 23 | 1, 2 |  | 2 |
|  | Flush the toilet | 16, 18, 19, 22, 23 |  | F, G | 1 |
|  | Use the bathroom independently (sitting on the toilet and standing up) | 12, 24, 25, 29, 32 | 27, 28 |  | 2, 10 |

| **Performing tasks/academic activities** | | **MFM-32** | **HFMSE** | **RULM** | **CHOP INTEND** |
| --- | --- | --- | --- | --- | --- |
|  | Turn the pages of a book | 17, 20, 21 |  | H, I | 1 |
|  | Read books/watch videos while lying down | 1, 2 | 17 |  | 4, 14 |
|  | Read books/watch videos while lying face down |  | 11, 12, 13 |  | 1, 15 |
|  | Keep a sitting position on a desk and chair | 13, 23 | 5, 6, 7, 8, 9 | B | 12 |
