# Supplementary figures and images for: Improvement in functional motor scores in patients with non-ambulatory spinal muscle atrophy during Nusinersen treatment in South Korea: a single center study
Source: BMC Neurol. 2024 Jun 20;24:210. doi: 10.1186/s12883-024-03725-w (PMC11188501; doi:10.1186/s12883-024-03725-w)

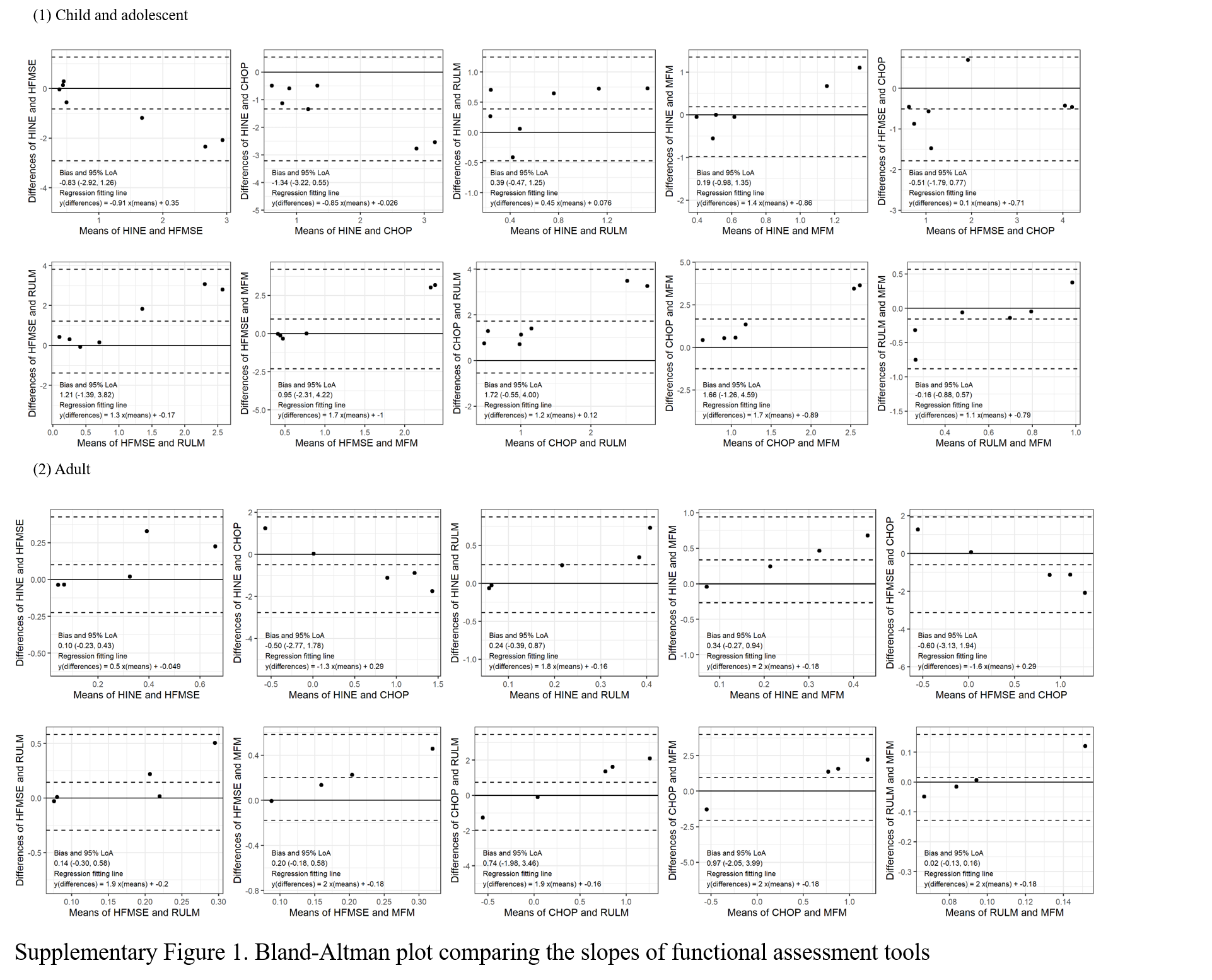

Supplement: Supplementary file 2 — Supplementary Material 2 [file 12883_2024_3725_MOESM2_ESM.png]
